# Remission of hypertension after treatment of giant simple renal cyst: a case report

**DOI:** 10.1186/1757-1626-2-9152

**Published:** 2009-12-07

**Authors:** Youness Ahallal, Abdelhak Khallouk, Mohammed Fadl Tazi, Elmehdi Tazi, Mohammed Jamal Elfassi, Moulay Hassan Farih

**Affiliations:** 1 Department of Urology, Teaching Hospital II, Fez, 30000, Morocco; 2 Department of Oncology, National Institut of Oncology, Teaching Hospital IbnSina, 1000, Rabat, Morocco

## Abstract

Renal cysts are common in old patients, and usually remain untreated. Giant renal cysts measuring more than 15 cm in greatest diameter are uncommon and the association with hypertension is very rare. We present a case of a 25-year-old woman with a giant right renal cyst associated with hypertension that was treated by laparoscopic excision, followed by resolution hypertension.

## Background

Renal cysts are acquired lesions of the kidney [1,2]. Although there is no accepted theory regarding the origin of renal cysts, at present, it is believed that they originate from the diverticulae of the distal convoluted or collecting tubules. Renal cysts are commonly an incidental ultrasound finding. It is generally believed to be a harmless anomaly. However, cases of complicated renal cysts have been reported [3]

## Case Presentation

**Figure 1:**
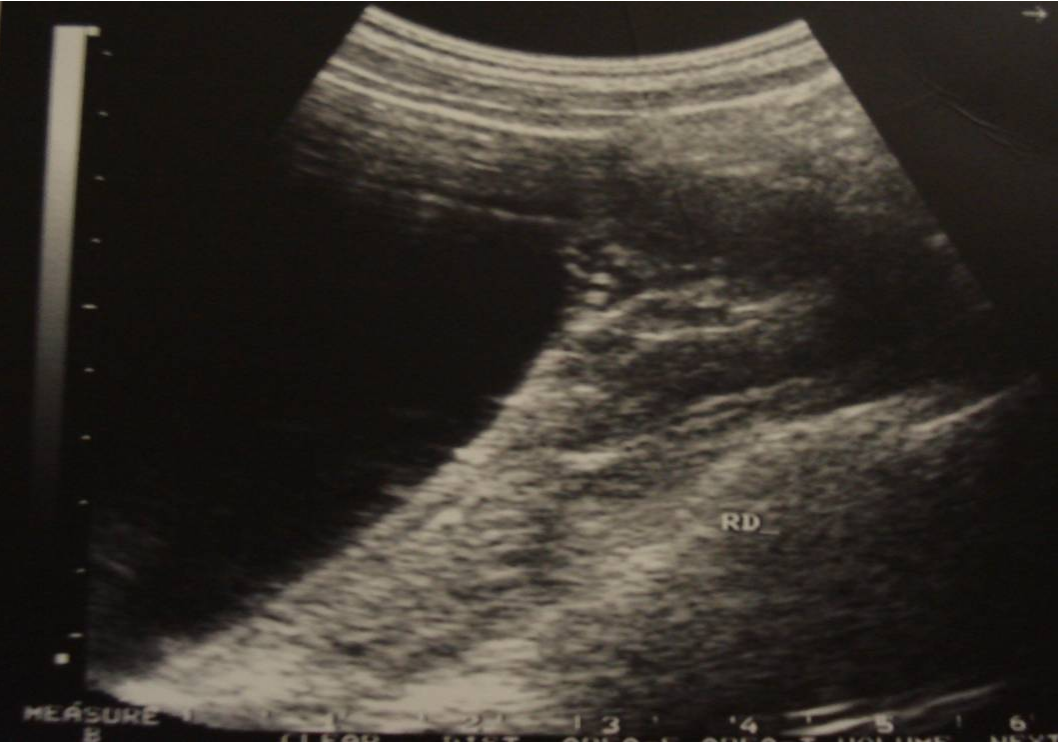
**Ultrasonography showing a giant right renal cyst**.

**Figure 2:**
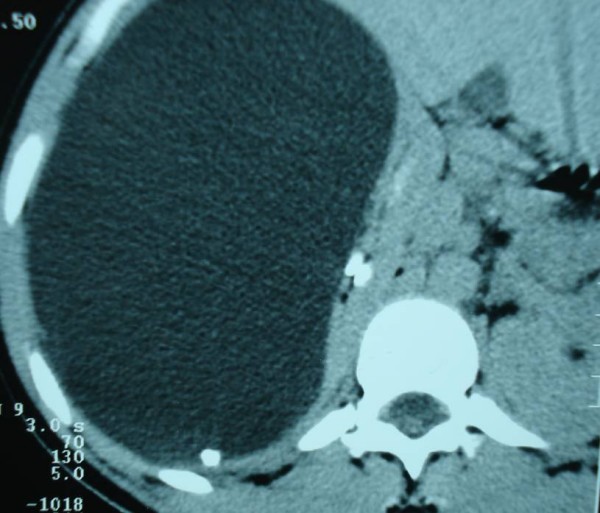
**CT scan showing a 15 cm size right renal cyst**.

The patient, a 25-year-old Moroccan housewife, gave a 5 years history of right lumbar pain. The physical examination revealed a non tender mass in the right flank and abdomen and hypertension (190/125 mm Hg), the rest of the physical examination was normal. Ultrasonography performed on the patient revealed a 15 cm size giant right renal cyst (figure 1) and an additional computed tomography of the abdomen demonstrated a simple right renal cyst measuring 17 × 20 cm, compressing the kidney (figure 2). The complete blood cell count, serum electrolytes, blood urea nitrogen, creatinine level and urinalysis were normal.

The cyst was managed by laparoscopic surgery with transperitoneal access. Surgery consisted of decompression and excision of the anterolateral wall of the cyst. Administration of indigocarmine indicated no communication between the renal collecting system and the cyst. Cytologicexamination of the cyst fluid for tumor was negative. Pathologic examination of the specimen revealed a non complicated simple renal cyst. Postoperatively, the patient was normotensive (115/75 mm Hg) without medication.

## Discussion

Simple renal cortical and parapelvic cysts are common and benign and usually require no treatment [3,4]. Occasionally the location and size of simple cysts can cause pain, collecting system obstruction, or hypertension. It is a common incidental radiographic and postmortem finding. It is estimated that evidence of renal cysts exists in 50% of the adult population. The large use of ultrasonography and CT produced an increase in the detection of renal cysts [4]. Simple renal cysts occur with an incidence of at least 20% by 40 years of age and 33% by 60 years. However, giant renal cysts measuring more than 15 cm rarely occur and association with secondary hypertension is uncommon [5].

Renin, a second renal peptide hormone, is stimulated when the extracellular fluid volume is reduced. Local renal ischemia, caused by cyst expansion, led to stimulation of the reninangiotensin-aldosterone system, which may explain the hypertension [5,6]. Renal cysts can be treated by percutaneous aspiration with or without injection of sclerosants [7,8], percutaneous marsupialization, open surgery and, most recently, by laparoscopic surgery with transperitoneal or retroperitoneal access. In our case, we did not choose aspiration and injection of sclerosants in order to have the cyst examined pathologically. Marsupialization using a laparoscopic technique is the best and the least invasive technique for such large renal cysts [3,5].

## Conclusion

Giant renal cysts causing hypertension are uncommon. Our case demonstrates that adequate cyst treatment can lead to hypertension remission.

## Consent

Written informed consent was obtained from the patient for publication of this case report and accompanying images. A copy of the written consent is available for review by the Editor-in-Chief of this journal.

## Competing interests

The authors declare that they have no competing interests.

## Authors' contributions

YA analyzed and interpreted the patient data regarding the retroperitoneal disease. MFT and ET have made contributions to conception and design, and acquisition of data. MJE and AK have been involved in drafting the manuscript and revising it critically for important intellectual content. MHF has given final approval of the version to be published.

All authors read and approved the final manuscript.
